# Bis{μ-2-[(pyridin-2-yl)imino­meth­yl]phenolato}bis­[(2-formyl­phenolato)copper(II)]

**DOI:** 10.1107/S1600536811015674

**Published:** 2011-05-07

**Authors:** Hui-Chang Chang, Jacqueline M. Cole, Tze-Chia Lin, Sven O. Sylvester, Paul G. Waddell

**Affiliations:** a Cavendish Laboratory, University of Cambridge, J. J. Thomson Avenue, Cambridge CB3 0HE, England

## Abstract

The asymmetric unit of the title compound, [Cu_2_(C_12_H_9_N_2_O)_2_(C_7_H_5_O_2_)_2_], contains two independent (2-formyl­phen­olato){2-[(pyridin-2-yl)imino­meth­yl]phenolato}copper(II) mol­ecules that form pseudocentrosymmetric dimers *via* inter­actions between the Cu and pyridyl N atoms of independent monomers. The square-planar geometry of the Cu atoms in the monomer thus becomes square-based pyramidal in the dimer. The crystal studied was an inversion twin, with unequal populations of 0.353 (17) and 0.647 (17).

## Related literature

For related structures containing the salicyl­aldehyde ligand, see: McKinnon *et al.* (1964 ▶); Hall *et al.* (1965 ▶). For a related structure containing the (2-pyridyl­salicylaldimine) ligand, see: Drummond & Wood (1972 ▶).
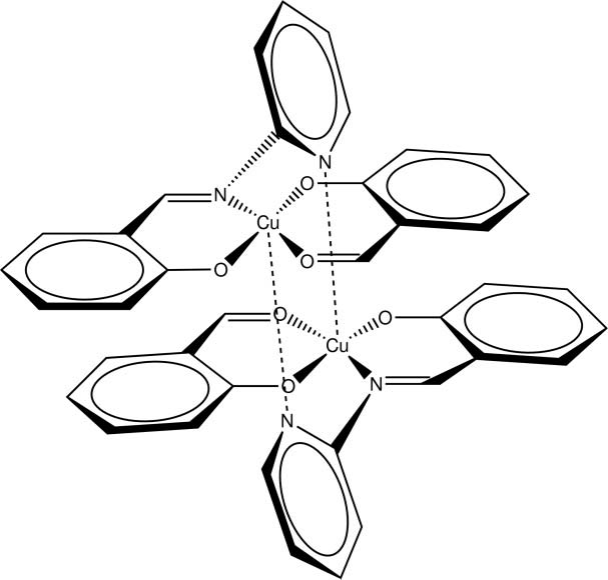


## Experimental

### 

#### Crystal data

[Cu_2_(C_12_H_9_N_2_O)_2_(C_7_H_5_O_2_)_2_]
*M* *_r_* = 763.72
Orthorhombic, 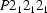

*a* = 8.9811 (18) Å
*b* = 18.856 (4) Å
*c* = 19.612 (4) Å
*V* = 3321.2 (12) Å^3^
*Z* = 4
Mo *K*α radiation
μ = 1.34 mm^−1^
*T* = 150 K
0.20 × 0.12 × 0.06 mm

#### Data collection

Rigaku Saturn724+ diffractometer
Absorption correction: multi-scan (*ABSCOR*; Higashi, 1995 ▶) *T*_min_ = 0.736, *T*_max_ = 1
33976 measured reflections
6782 independent reflections
6545 reflections with *I* > 2σ(*I*)
*R*_int_ = 0.076

#### Refinement

*R*[*F* ^2^ > 2σ(*F* ^2^)] = 0.057
*wR*(*F* ^2^) = 0.106
*S* = 1.20
6782 reflections
452 parameters
H-atom parameters constrained
Δρ_max_ = 0.39 e Å^−3^
Δρ_min_ = −0.41 e Å^−3^
Absolute structure: Flack (1983 ▶), 2964 Friedel pairs
Flack parameter: 0.353 (17)

### 

Data collection: *CrystalClear* (Rigaku, 2008 ▶); cell refinement: *CrystalClear*; data reduction: *CrystalClear*; program(s) used to solve structure: *SHELXS97* (Sheldrick, 2008 ▶); program(s) used to refine structure: *SHELXL97* (Sheldrick, 2008 ▶); molecular graphics: *SHELXTL* (Sheldrick, 2008 ▶); software used to prepare material for publication: *WinGX* (Farrugia, 1999 ▶).

## Supplementary Material

Crystal structure: contains datablocks global, I. DOI: 10.1107/S1600536811015674/pk2308sup1.cif

Structure factors: contains datablocks I. DOI: 10.1107/S1600536811015674/pk2308Isup2.hkl

Additional supplementary materials:  crystallographic information; 3D view; checkCIF report

## Figures and Tables

**Fig. 1.**
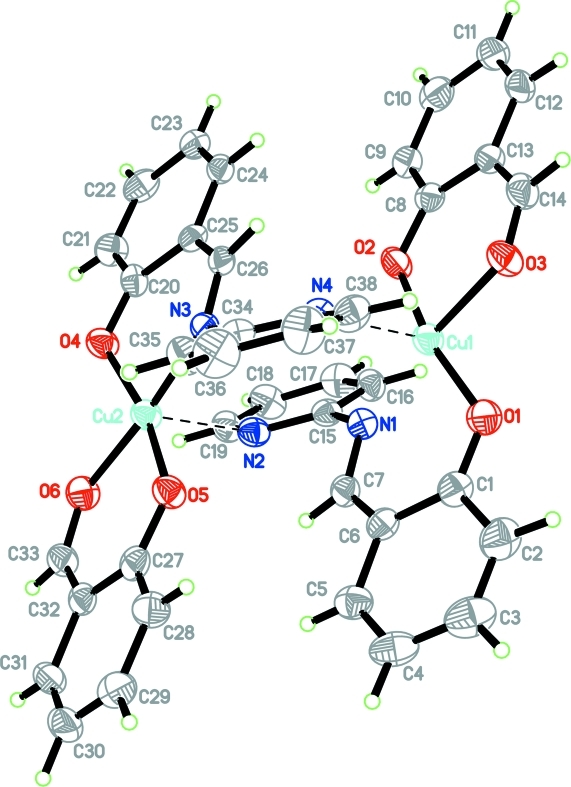
The structure of the asymmetric unit of the title compound with displacement ellipsoids drawn at the 50% probability level.

## supplementary crystallographic information

### Comment

The title compound forms dimers in which the monomer units are related by a non-crystallographic inversion operation. Intermolecular contacts are present between the copper(II) atoms and pyridyl nitrogen atoms (2.482 (4)Å and 2.469 (4) Å) with angles between the Cu—N contact and the plane of the pyridyl group observed to be 157.1 (5)° and 150.4 (5)°. These contacts appear to be the predominant cause of dimer formation since there is no evidence for any other significant intermolecular interactions.  The bonding between the salicylaldehyde ligand and the copper atom can be compared to the two known polymorphs of homoleptic bissalicylaldehydatocopper(II), one described with the symmetry of P2_1_/n (McKinnon *et al.*, 1964), and the other as P2_1_/c (Hall *et al.*, 1965). The Cu—O bonds Cu1—O2 and Cu2—O5 in the title compound are observed to be 1.917 (3)Å and 1.918 (3)Å respectively, which is longer than the values of the equivalent bonds in the two polymorphs of bissalicylaldehydatocopper(II) where Cu—O1 is 1.86Å in the P2_1_/n polymorph and 1.90Å in the P2_1_/c polymorph. The Cu—O bonds Cu1—O3 and Cu2—O6 in the title compound are observed to be 2.022 (3)Å and 2.019 (3)Å respectively, also longer than the values of the equivalent bonds in the two polymorphs of bissalicylaldehydatocopper(II) where Cu—O2 is 1.98Å in the P2_1_/n polymorph and 1.94Å in the P2_1_/c polymorph. Additionally, the ligand bite-angles O2—Cu1—O3 and O5—Cu2—O6 are observed to be 91.05 (14)° and 90.89 (14)° respectively, lower than the equivalent angle O1—Cu—O2 in both of the known polymorphs of bissalicylaldehydatocopper(II), which are observed to be 94.8° in the P2_1_/n polymorph and 95° in the P2_1_/c polymorph. Bond distances from the copper atom to the 2-pyridylsalicylaldimine ligand are observed to be smaller than observed in a (2-pyridylsalicylaldimine)copper(I) tetramer (Drummond *et al.*, 1972).  In the title compound the bond lengths Cu1—O1 and Cu2—O4 are observed to be 1.916 (4)Å and 1.914 (3)Å respectively, shorter than the equivalent bond length Cu1—O1 in the (2-pyridylsalicylaldimine) copper(I) tetramer which is observed to be 1.965 (6) Å. The Cu1—N1 and Cu2—N3 bond lengths in the title compound, which are observed to be 2.022 (4)Å and 2.022 (4)Å respectively, can be compared to the equivalent bond length Cu1—N1 in the (2-pyridylsalicylaldimine) copper(I) tetramer, which is observed to be 1.975 (8) Å, shorter than those observed in the title compound. These differences can be attributed to the lower oxidation state of copper in the (2-pyridylsalicylaldimine) copper(I) tetramer compared to that in the title compound.

### Experimental

A suspension of bis(2-pyridylsalicylaldimine)copper(II) (1 mg, 0.0022 mmol) in methanol (*ca*. 3 ml) was heated to *ca*. 323 K until fully dissolved. The solution was then allowed to cool to room temperature. Crystals suitable for single-crystal X-ray crystallography were grown *via* slow evaporation of methanol over seven days.

### Crystal data

**Table:**

[Cu_2_(C_12_H_9_N_2_O)_2_(C_7_H_5_O_2_)_2_]	*F*(000) = 1560
*M**_r_* = 763.72	*D*_x_ = 1.527 Mg m^−^^3^
Orthorhombic, *P*2_1_2_1_2_1_	Mo *K*α radiation, λ = 0.71073 Å
Hall symbol: P 2ac 2ab	Cell parameters from 9105 reflections
*a* = 8.9811 (18) Å	θ = 2.1–30.4°
*b* = 18.856 (4) Å	µ = 1.34 mm^−^^1^
*c* = 19.612 (4) Å	*T* = 150 K
*V* = 3321.2 (12) Å^3^	Prism, yellow
*Z* = 4	0.20 × 0.12 × 0.06 mm

### Data collection

**Table:**

Rigaku Saturn724+ diffractometer	6782 independent reflections
graphite	6545 reflections with *I* > 2σ(*I*)
Detector resolution: 28.6 pixels mm^-1^	*R*_int_ = 0.076
ω scans	θ_max_ = 26.4°, θ_min_ = 3.1°
Absorption correction: multi-scan (*ABSCOR*; Higashi, 1995)	*h* = −11→10
*T*_min_ = 0.736, *T*_max_ = 1	*k* = −23→22
33976 measured reflections	*l* = −23→24

### Refinement

**Table:**

Refinement on *F*^2^	Secondary atom site location: difference Fourier map
Least-squares matrix: full	Hydrogen site location: inferred from neighbouring sites
*R*[*F*^2^ > 2σ(*F*^2^)] = 0.057	H-atom parameters constrained
*wR*(*F*^2^) = 0.106	*w* = 1/[σ^2^(*F*_o_^2^) + (0.0194*P*)^2^ + 4.0051*P*] where *P* = (*F*_o_^2^ + 2*F*_c_^2^)/3
*S* = 1.20	(Δ/σ)_max_ = 0.001
6782 reflections	Δρ_max_ = 0.39 e Å^−^^3^
452 parameters	Δρ_min_ = −0.41 e Å^−^^3^
0 restraints	Absolute structure: Flack (1983), 2964 Friedel pairs
Primary atom site location: structure-invariant direct methods	Flack parameter: 0.353 (17)

### Special details

**Table:**

Geometry. All s.u.'s (except the s.u. in the dihedral angle between two l.s. planes) are estimated using the full covariance matrix. The cell s.u.'s are taken into account individually in the estimation of s.u.'s in distances, angles and torsion angles; correlations between s.u.'s in cell parameters are only used when they are defined by crystal symmetry. An approximate (isotropic) treatment of cell s.u.'s is used for estimating s.u.'s involving l.s. planes.
Refinement. Refinement of *F*^2^ against ALL reflections. The weighted *R*-factor *wR* and goodness of fit *S* are based on *F*^2^, conventional *R*-factors *R* are based on *F*, with *F* set to zero for negative *F*^2^. The threshold expression of *F*^2^ > 2σ(*F*^2^) is used only for calculating *R*-factors(gt) *etc*. and is not relevant to the choice of reflections for refinement. *R*-factors based on *F*^2^ are statistically about twice as large as those based on *F*, and *R*- factors based on ALL data will be even larger.

### Fractional atomic coordinates and isotropic or equivalent isotropic displacement parameters (Å^2^)

**Table:**

	*x*	*y*	*z*	*U*_iso_*/*U*_eq_	
Cu1	0.18124 (6)	0.99821 (3)	0.80722 (3)	0.02880 (13)	
Cu2	0.34241 (6)	0.77028 (3)	0.70000 (3)	0.02792 (13)	
O2	0.0380 (4)	0.99272 (19)	0.73492 (16)	0.0324 (8)	
N3	0.1713 (5)	0.80580 (19)	0.75822 (18)	0.0234 (8)	
N1	0.3472 (5)	0.9601 (2)	0.74793 (18)	0.0254 (8)	
O4	0.2110 (4)	0.74573 (18)	0.62687 (16)	0.0323 (8)	
O6	0.5021 (4)	0.7284 (2)	0.63978 (16)	0.0347 (8)	
C31	0.8313 (6)	0.6798 (3)	0.7383 (3)	0.0348 (11)	
H31	0.8822	0.6595	0.7019	0.042*	
O1	0.3198 (4)	1.02568 (18)	0.87665 (17)	0.0375 (9)	
O3	0.0291 (4)	1.0429 (2)	0.86985 (17)	0.0354 (8)	
C1	0.4496 (5)	0.9962 (3)	0.8891 (2)	0.0322 (11)	
O5	0.4773 (4)	0.77314 (19)	0.77578 (15)	0.0312 (7)	
C17	0.2743 (6)	0.9990 (3)	0.5662 (2)	0.0391 (12)	
H17	0.2379	1.0355	0.539	0.047*	
C25	0.0007 (5)	0.8201 (3)	0.6601 (2)	0.0258 (10)	
C15	0.3331 (6)	0.9506 (2)	0.6754 (2)	0.0270 (10)	
C20	0.0858 (5)	0.7781 (3)	0.6120 (2)	0.0272 (10)	
C30	0.8958 (6)	0.6803 (3)	0.8016 (3)	0.0407 (12)	
H30	0.9898	0.6608	0.8083	0.049*	
C21	0.0235 (6)	0.7718 (3)	0.5450 (2)	0.0348 (11)	
H21	0.0741	0.7453	0.5124	0.042*	
C13	−0.1640 (6)	1.0600 (2)	0.7855 (2)	0.0300 (10)	
C3	0.6615 (7)	0.9818 (3)	0.9673 (3)	0.0501 (15)	
H3	0.708	0.9937	1.0081	0.06*	
C33	0.6283 (6)	0.7067 (3)	0.6591 (3)	0.0343 (12)	
H33	0.6893	0.6868	0.626	0.041*	
C27	0.6080 (5)	0.7423 (3)	0.7829 (3)	0.0309 (11)	
C28	0.6772 (6)	0.7409 (3)	0.8474 (2)	0.0366 (11)	
H28	0.6281	0.7604	0.8847	0.044*	
C18	0.3240 (6)	0.9365 (3)	0.5366 (2)	0.0391 (12)	
H18	0.3243	0.9307	0.4895	0.047*	
C6	0.5217 (5)	0.9478 (3)	0.8436 (2)	0.0287 (11)	
C7	0.4716 (6)	0.9365 (3)	0.7743 (2)	0.0302 (11)	
H7	0.5333	0.9101	0.746	0.036*	
C2	0.5278 (6)	1.0136 (3)	0.9505 (3)	0.0443 (14)	
H2	0.4875	1.0472	0.9799	0.053*	
C26	0.0461 (5)	0.8277 (2)	0.7302 (2)	0.0247 (10)	
H26	−0.0205	0.8507	0.7589	0.03*	
C16	0.2791 (5)	1.0069 (3)	0.6366 (2)	0.0318 (11)	
H16	0.2473	1.0487	0.6573	0.038*	
C5	0.6610 (6)	0.9168 (3)	0.8619 (3)	0.0353 (11)	
H5	0.7075	0.8858	0.8317	0.042*	
C32	0.6868 (6)	0.7099 (2)	0.7272 (2)	0.0276 (10)	
C14	−0.0977 (6)	1.0650 (3)	0.8514 (3)	0.0355 (12)	
H14	−0.1545	1.0871	0.8849	0.043*	
N4	0.1434 (4)	0.8785 (2)	0.85692 (18)	0.0282 (9)	
C23	−0.1931 (6)	0.8431 (3)	0.5754 (2)	0.0316 (11)	
H23	−0.2834	0.8638	0.5633	0.038*	
N2	0.3766 (4)	0.8885 (2)	0.64818 (19)	0.0294 (9)	
C24	−0.1378 (5)	0.8501 (2)	0.6410 (2)	0.0267 (10)	
H24	−0.1929	0.8749	0.6733	0.032*	
C38	0.1394 (6)	0.8842 (3)	0.9252 (2)	0.0347 (12)	
H38	0.1143	0.9279	0.9441	0.042*	
C29	0.8168 (6)	0.7110 (3)	0.8563 (3)	0.0374 (12)	
H29	0.8595	0.7112	0.8995	0.045*	
C9	−0.1666 (6)	1.0256 (2)	0.6661 (2)	0.0330 (11)	
H9	−0.1206	1.0052	0.6283	0.04*	
C10	−0.3053 (6)	1.0558 (3)	0.6585 (3)	0.0423 (13)	
H10	−0.3515	1.0543	0.6161	0.051*	
C34	0.1807 (5)	0.8147 (2)	0.8304 (2)	0.0261 (10)	
C22	−0.1100 (6)	0.8042 (3)	0.5273 (3)	0.0379 (13)	
H22	−0.1452	0.8	0.4829	0.045*	
C19	0.3730 (5)	0.8830 (3)	0.5794 (2)	0.0322 (11)	
H19	0.4054	0.8408	0.5597	0.039*	
C36	0.2157 (7)	0.7636 (3)	0.9410 (3)	0.0461 (15)	
H36	0.2403	0.7257	0.9691	0.055*	
C35	0.2229 (6)	0.7568 (3)	0.8709 (2)	0.0340 (12)	
H35	0.2551	0.7148	0.851	0.041*	
C8	−0.0916 (5)	1.0247 (2)	0.7303 (2)	0.0269 (10)	
C4	0.7286 (6)	0.9319 (3)	0.9240 (3)	0.0433 (14)	
H4	0.8166	0.9094	0.9366	0.052*	
C11	−0.3798 (6)	1.0892 (3)	0.7140 (3)	0.0407 (14)	
H11	−0.474	1.1089	0.7087	0.049*	
C12	−0.3068 (6)	1.0911 (2)	0.7758 (3)	0.0378 (13)	
H12	−0.3525	1.1136	0.8124	0.045*	
C37	0.1707 (7)	0.8285 (3)	0.9690 (2)	0.0434 (13)	
H37	0.1621	0.8341	1.016	0.052*	

### Atomic displacement parameters (Å^2^)

**Table:**

	*U*^11^	*U*^22^	*U*^33^	*U*^12^	*U*^13^	*U*^23^
Cu1	0.0285 (3)	0.0316 (3)	0.0263 (3)	0.0020 (3)	0.0013 (3)	−0.0047 (3)
Cu2	0.0274 (3)	0.0311 (3)	0.0253 (3)	0.0031 (2)	0.0006 (3)	−0.0039 (2)
O2	0.0307 (18)	0.0324 (19)	0.0341 (18)	0.0088 (16)	−0.0011 (14)	−0.0055 (16)
N3	0.023 (2)	0.0243 (19)	0.0230 (18)	−0.0007 (17)	0.0021 (18)	−0.0017 (15)
N1	0.028 (2)	0.026 (2)	0.0216 (18)	−0.0002 (18)	0.0025 (18)	0.0029 (15)
O4	0.0283 (18)	0.038 (2)	0.0305 (18)	0.0045 (15)	−0.0007 (14)	−0.0087 (15)
O6	0.0349 (19)	0.039 (2)	0.0298 (18)	0.0015 (17)	0.0035 (15)	−0.0071 (17)
C31	0.028 (3)	0.028 (3)	0.049 (3)	0.005 (2)	0.006 (3)	0.006 (2)
O1	0.034 (2)	0.044 (2)	0.0344 (19)	0.0005 (17)	0.0016 (17)	−0.0126 (15)
O3	0.034 (2)	0.041 (2)	0.0313 (19)	0.0075 (17)	0.0045 (16)	−0.0032 (16)
C1	0.034 (3)	0.038 (3)	0.024 (2)	−0.003 (2)	0.0014 (19)	−0.003 (2)
O5	0.0316 (17)	0.0340 (19)	0.0280 (17)	0.0060 (16)	−0.0017 (14)	−0.0036 (15)
C17	0.040 (3)	0.042 (3)	0.035 (3)	−0.001 (3)	0.000 (2)	0.012 (3)
C25	0.025 (2)	0.028 (3)	0.025 (2)	−0.0067 (19)	0.0025 (19)	−0.0025 (19)
C15	0.024 (2)	0.033 (2)	0.025 (2)	−0.002 (2)	0.003 (2)	−0.0017 (18)
C20	0.034 (3)	0.024 (2)	0.024 (2)	0.001 (2)	0.006 (2)	0.001 (2)
C30	0.034 (3)	0.028 (3)	0.060 (3)	0.005 (2)	−0.001 (3)	0.010 (3)
C21	0.036 (3)	0.043 (3)	0.025 (2)	0.000 (2)	−0.003 (2)	−0.007 (2)
C13	0.026 (2)	0.025 (2)	0.039 (3)	−0.001 (2)	0.002 (2)	0.0011 (19)
C3	0.045 (3)	0.073 (4)	0.032 (3)	−0.009 (3)	−0.005 (3)	0.001 (3)
C33	0.031 (3)	0.032 (3)	0.040 (3)	0.002 (2)	0.008 (2)	−0.005 (2)
C27	0.033 (3)	0.022 (2)	0.037 (3)	−0.0062 (19)	0.004 (2)	0.004 (2)
C28	0.036 (3)	0.038 (3)	0.036 (3)	0.001 (3)	−0.003 (2)	0.001 (2)
C18	0.042 (3)	0.051 (3)	0.024 (2)	0.006 (3)	0.003 (3)	−0.002 (2)
C6	0.029 (3)	0.028 (3)	0.029 (2)	−0.005 (2)	0.001 (2)	0.002 (2)
C7	0.030 (3)	0.023 (2)	0.037 (3)	−0.002 (2)	0.005 (2)	0.001 (2)
C2	0.048 (3)	0.055 (4)	0.030 (3)	−0.004 (3)	−0.001 (2)	−0.010 (3)
C26	0.025 (2)	0.023 (2)	0.026 (2)	−0.0013 (19)	0.0034 (19)	−0.0048 (19)
C16	0.034 (3)	0.027 (3)	0.035 (2)	0.000 (2)	0.006 (2)	0.001 (2)
C5	0.031 (3)	0.037 (3)	0.037 (3)	−0.002 (2)	−0.001 (3)	0.007 (2)
C32	0.029 (2)	0.021 (2)	0.033 (2)	−0.0026 (19)	0.006 (2)	0.0026 (18)
C14	0.038 (3)	0.031 (3)	0.037 (3)	0.000 (2)	0.014 (2)	−0.002 (2)
N4	0.030 (2)	0.031 (2)	0.0240 (18)	−0.0013 (17)	0.0035 (17)	0.0006 (16)
C23	0.029 (3)	0.031 (3)	0.035 (3)	−0.002 (2)	−0.003 (2)	−0.002 (2)
N2	0.027 (2)	0.035 (2)	0.027 (2)	0.0006 (17)	0.0028 (16)	−0.0009 (18)
C24	0.028 (3)	0.024 (2)	0.028 (2)	−0.0013 (19)	0.005 (2)	−0.0018 (19)
C38	0.036 (3)	0.041 (3)	0.027 (2)	−0.004 (2)	−0.006 (2)	−0.005 (2)
C29	0.037 (3)	0.034 (3)	0.040 (3)	−0.004 (2)	−0.009 (3)	0.012 (2)
C9	0.033 (3)	0.029 (2)	0.037 (3)	−0.002 (2)	0.001 (2)	−0.0013 (19)
C10	0.040 (3)	0.031 (3)	0.056 (3)	−0.002 (2)	−0.014 (3)	0.007 (2)
C34	0.022 (2)	0.032 (2)	0.024 (2)	−0.003 (2)	−0.001 (2)	0.0017 (18)
C22	0.039 (3)	0.046 (3)	0.028 (3)	−0.011 (2)	−0.006 (2)	−0.001 (2)
C19	0.033 (3)	0.036 (3)	0.028 (2)	0.002 (2)	0.003 (2)	−0.003 (2)
C36	0.059 (4)	0.050 (4)	0.029 (3)	0.000 (3)	−0.010 (3)	0.013 (3)
C35	0.038 (3)	0.034 (3)	0.029 (2)	0.005 (2)	0.000 (2)	0.002 (2)
C8	0.026 (2)	0.022 (2)	0.032 (3)	−0.0022 (18)	0.001 (2)	0.0059 (19)
C4	0.034 (3)	0.063 (4)	0.034 (3)	−0.004 (3)	−0.009 (2)	0.008 (3)
C11	0.033 (3)	0.027 (3)	0.062 (4)	0.004 (2)	−0.004 (3)	0.004 (3)
C12	0.034 (3)	0.021 (2)	0.059 (3)	0.002 (2)	0.010 (3)	0.002 (2)
C37	0.055 (4)	0.049 (3)	0.026 (2)	−0.001 (3)	−0.002 (3)	0.000 (2)

### Geometric parameters (Å, °)

**Table:**

Cu1—O1	1.916 (4)	C33—H33	0.93
Cu1—O2	1.917 (3)	C27—C28	1.410 (7)
Cu1—O3	2.022 (3)	C27—C32	1.437 (6)
Cu1—N1	2.022 (4)	C28—C29	1.386 (7)
Cu2—O4	1.914 (3)	C28—H28	0.93
Cu2—O5	1.918 (3)	C18—C19	1.384 (7)
Cu2—O6	2.019 (3)	C18—H18	0.93
Cu2—N3	2.028 (4)	C6—C5	1.427 (7)
O2—C8	1.315 (6)	C6—C7	1.447 (6)
N3—C26	1.318 (6)	C7—H7	0.93
N3—C34	1.429 (5)	C2—H2	0.93
N1—C7	1.309 (6)	C26—H26	0.93
N1—C15	1.439 (5)	C16—H16	0.93
O4—C20	1.312 (6)	C5—C4	1.390 (7)
O6—C33	1.262 (6)	C5—H5	0.93
C31—C30	1.369 (7)	C14—H14	0.93
C31—C32	1.433 (7)	N4—C38	1.345 (6)
C31—H31	0.93	N4—C34	1.352 (6)
O1—C1	1.315 (6)	C23—C24	1.386 (6)
O3—C14	1.265 (6)	C23—C22	1.410 (7)
C1—C6	1.432 (7)	C23—H23	0.93
C1—C2	1.432 (7)	N2—C19	1.354 (6)
O5—C27	1.318 (6)	C24—H24	0.93
C17—C18	1.388 (8)	C38—C37	1.386 (7)
C17—C16	1.390 (6)	C38—H38	0.93
C17—H17	0.93	C29—H29	0.93
C25—C24	1.417 (7)	C9—C10	1.378 (7)
C25—C26	1.441 (6)	C9—C8	1.429 (6)
C25—C20	1.448 (6)	C9—H9	0.93
C15—N2	1.345 (6)	C10—C11	1.423 (8)
C15—C16	1.394 (7)	C10—H10	0.93
C20—C21	1.434 (6)	C34—C35	1.402 (6)
C30—C29	1.412 (8)	C22—H22	0.93
C30—H30	0.93	C19—H19	0.93
C21—C22	1.390 (7)	C36—C35	1.383 (7)
C21—H21	0.93	C36—C37	1.401 (8)
C13—C12	1.424 (7)	C36—H36	0.93
C13—C14	1.426 (7)	C35—H35	0.93
C13—C8	1.427 (6)	C4—H4	0.93
C3—C2	1.382 (8)	C11—C12	1.379 (7)
C3—C4	1.403 (8)	C11—H11	0.93
C3—H3	0.93	C12—H12	0.93
C33—C32	1.437 (6)	C37—H37	0.93
			
O1—Cu1—O2	167.40 (15)	N1—C7—H7	117
O1—Cu1—O3	83.95 (14)	C6—C7—H7	117
O2—Cu1—O3	91.05 (14)	C3—C2—C1	121.8 (5)
O1—Cu1—N1	91.50 (16)	C3—C2—H2	119.1
O2—Cu1—N1	92.86 (14)	C1—C2—H2	119.1
O3—Cu1—N1	174.69 (16)	N3—C26—C25	127.5 (4)
O4—Cu2—O5	167.61 (15)	N3—C26—H26	116.3
O4—Cu2—O6	84.55 (13)	C25—C26—H26	116.3
O5—Cu2—O6	90.89 (14)	C17—C16—C15	118.1 (5)
O4—Cu2—N3	91.98 (15)	C17—C16—H16	120.9
O5—Cu2—N3	91.89 (14)	C15—C16—H16	120.9
O6—Cu2—N3	175.40 (15)	C4—C5—C6	121.3 (5)
C8—O2—Cu1	128.3 (3)	C4—C5—H5	119.3
C26—N3—C34	115.3 (4)	C6—C5—H5	119.3
C26—N3—Cu2	121.0 (3)	C31—C32—C33	117.2 (4)
C34—N3—Cu2	123.5 (3)	C31—C32—C27	119.9 (4)
C7—N1—C15	115.0 (4)	C33—C32—C27	122.9 (5)
C7—N1—Cu1	121.5 (3)	O3—C14—C13	127.8 (5)
C15—N1—Cu1	123.2 (3)	O3—C14—H14	116.1
C20—O4—Cu2	125.6 (3)	C13—C14—H14	116.1
C33—O6—Cu2	126.0 (3)	C38—N4—C34	117.4 (4)
C30—C31—C32	121.2 (5)	C24—C23—C22	118.8 (5)
C30—C31—H31	119.4	C24—C23—H23	120.6
C32—C31—H31	119.4	C22—C23—H23	120.6
C1—O1—Cu1	126.4 (3)	C15—N2—C19	117.1 (4)
C14—O3—Cu1	124.9 (3)	C23—C24—C25	121.5 (4)
O1—C1—C6	123.7 (4)	C23—C24—H24	119.3
O1—C1—C2	119.6 (5)	C25—C24—H24	119.3
C6—C1—C2	116.7 (5)	N4—C38—C37	123.4 (5)
C27—O5—Cu2	129.2 (3)	N4—C38—H38	118.3
C18—C17—C16	119.8 (5)	C37—C38—H38	118.3
C18—C17—H17	120.1	C28—C29—C30	121.7 (5)
C16—C17—H17	120.1	C28—C29—H29	119.2
C24—C25—C26	117.4 (4)	C30—C29—H29	119.2
C24—C25—C20	120.6 (4)	C10—C9—C8	121.6 (5)
C26—C25—C20	121.7 (4)	C10—C9—H9	119.2
N2—C15—C16	123.2 (4)	C8—C9—H9	119.2
N2—C15—N1	118.4 (4)	C9—C10—C11	121.8 (5)
C16—C15—N1	118.4 (4)	C9—C10—H10	119.1
O4—C20—C21	119.9 (4)	C11—C10—H10	119.1
O4—C20—C25	124.2 (4)	N4—C34—C35	122.8 (4)
C21—C20—C25	115.8 (4)	N4—C34—N3	118.1 (4)
C31—C30—C29	118.6 (5)	C35—C34—N3	119.0 (4)
C31—C30—H30	120.7	C21—C22—C23	121.2 (5)
C29—C30—H30	120.7	C21—C22—H22	119.4
C22—C21—C20	122.0 (5)	C23—C22—H22	119.4
C22—C21—H21	119	N2—C19—C18	123.8 (5)
C20—C21—H21	119	N2—C19—H19	118.1
C12—C13—C14	118.1 (5)	C18—C19—H19	118.1
C12—C13—C8	120.0 (5)	C35—C36—C37	119.0 (5)
C14—C13—C8	121.9 (5)	C35—C36—H36	120.5
C2—C3—C4	121.4 (5)	C37—C36—H36	120.5
C2—C3—H3	119.3	C36—C35—C34	118.5 (5)
C4—C3—H3	119.3	C36—C35—H35	120.7
O6—C33—C32	126.5 (5)	C34—C35—H35	120.7
O6—C33—H33	116.8	O2—C8—C13	124.4 (4)
C32—C33—H33	116.8	O2—C8—C9	118.9 (4)
O5—C27—C28	119.7 (4)	C13—C8—C9	116.7 (4)
O5—C27—C32	123.1 (4)	C5—C4—C3	118.7 (5)
C28—C27—C32	117.2 (5)	C5—C4—H4	120.7
C29—C28—C27	121.4 (5)	C3—C4—H4	120.7
C29—C28—H28	119.3	C12—C11—C10	117.4 (5)
C27—C28—H28	119.3	C12—C11—H11	121.3
C19—C18—C17	117.9 (4)	C10—C11—H11	121.3
C19—C18—H18	121	C11—C12—C13	122.4 (5)
C17—C18—H18	121	C11—C12—H12	118.8
C5—C6—C1	120.0 (4)	C13—C12—H12	118.8
C5—C6—C7	116.7 (5)	C38—C37—C36	118.5 (5)
C1—C6—C7	122.6 (5)	C38—C37—H37	120.7
N1—C7—C6	125.9 (5)	C36—C37—H37	120.7
